# Glucose-6-phosphate dehydrogenase activity measured by spectrophotometry and associated genetic variants from the Oromiya zone, Ethiopia

**DOI:** 10.1186/s12936-018-2510-3

**Published:** 2018-10-12

**Authors:** Nora Kießling, Joaquin Brintrup, Ahmed Zeynudin, Nuredin Abduselam, Sylvia Götz, Margith Mack, Michael Pritsch, Andreas Wieser, Elisabeth Kohne, Nicole Berens-Riha

**Affiliations:** 1 Division of Infectious Diseases and Tropical Medicine, Medical Center of the University of Munich (LMU), Leopoldstrasse 5, 80802 Munich, Germany; 2 Hemoglobin Laboratory, Department of Pediatrics, University Hospital Ulm, Eythstrasse 24, 89075 Ulm, Germany; 3 Department of Medical Laboratory Sciences, Jimma University, Jimma, Ethiopia; 4 German Centre for Infection Research (DZIF) at LMU, Munich, Germany

## Abstract

**Background:**

The study aimed to gain first data on the prevalence of G6PD enzyme deficiency measured by spectrophotometry and associated genetic variants in Jimma and surroundings, Ethiopia. The area is a *Plasmodium vivax* endemic region, but 8-aminoquinolines such as primaquine are not recommended as G6PD testing is not available.

**Methods:**

Healthy volunteers were recruited at Jimma University, Ethiopia. Enzyme activity was tested by spectrophotometry at the University of Ulm, Germany. A G6PD RDT (Binax NOW^®^ G6PD, Alere, USA) was additionally performed. The *G6PD* gene was analysed for polymorphisms in a sub-population. Tests for haemoglobinopathies and the presence of malaria parasites were conducted.

**Results:**

No severe or moderate (cut-off 60%) G6PD deficiency was found in 206 volunteers. Median male activity was 6.1 U/g Hb. Eleven participants (5.4%) showed activities between 70 and 80%. No haemoglobinopathy was detected. None of the subjects showed asymptomatic parasitaemia. One G6PD-A+ variant (A376G) and one new non-synonymous mutation (G445A) were found.

**Conclusions:**

As the prevalence of G6PD deficiency seems low in this area, the use of 8-aminoquinolines should be encouraged. However, reliable G6PD testing methods have to be implemented and safe cut-off levels need to be defined.

**Electronic supplementary material:**

The online version of this article (10.1186/s12936-018-2510-3) contains supplementary material, which is available to authorized users.

## Background

Malaria is endemic in 75% of Ethiopia. For this parasitic disease, the predominant species is *Plasmodium falciparum* followed by *Plasmodium vivax* with regional variations. In Jimma zone, Southwestern Ethiopia, transmission is seasonal and unstable with a peak of infection after the rainy season in autumn. A study conducted by this group showed that 36–70% of the malaria infections in Jimma zone were caused by *P. vivax*, depending on the study location [1].

After the worldwide implementation of artemisinin-based-combination therapy (ACT), a general decline in falciparum malaria and a relative increase of vivax malaria was reported [2]. For a long time, *P. vivax* malaria was considered a harmless form of malaria compared to *P. falciparum* [3]. However, over the past few years, an increase of fatal and severe disease outcomes were described [4]. As a single vivax infection can result in multiple episodes due to relapses caused by the liver stages (hypnozoites), treatment of the liver stage is essential for controlling the disease. Additionally, increasing resistance of *P. falciparum* to ACT resulted in a containment strategy including the treatment of gametocytes to stop spread of resistance [5]. This strategy goes along with elimination efforts. The only recommended treatment by the World Health Organization (WHO) against *P. vivax* hypnozoites and *P. falciparum* gametocytes is the 8-aminoquinoline primaquine (PQ). A full 14-day course of primaquine (0.25 mg/kg/day) is required for radical cure of *P. vivax*, whereas a single-low-dose regimen is recommended for the elimination of *P. falciparum* gametocytes. The United States Food and Drug Administration (FDA) has now approved, under priority review, a single-dose regimen of tafenoquine for the radical cure of *P. vivax* in patients ≥ 16 years. Tafenoquine is a long-acting analogue of primaquine and as such an 8-aminoquinoline as well [6]. Unfortunately, the use of 8-aminoquinolines is compromised by the potential haemolysis in individuals with the enzyme deficiency of glucose-6-phosphate dehydrogenase (G6PD). In consequence, enzyme activity testing of G6PD is essential before the administration of 8-aminoquinolines.

Affecting over 400 million people, it is one of the most common enzyme deficiencies in the world that is probably protective against malaria especially severe malaria outcomes [7]. G6PD is an essential enzyme in the pentose phosphate pathway assuring normal oxidizing processes in the red blood cells (RBC). Although the inherited enzyme deficiency is mainly asymptomatic, it can cause neonatal hyperbilirubinaemia and chronical anaemia. The exposure to oxidative stress can provoke destruction of red blood cells and haemolysis, triggered by even small amounts of oxidants like primaquine, dapsone, paracetamol, fava beans and many more [8].

The size of the *G6PD* gene is 18.5 kB. It consists of 13 exons, 12 introns and is located on the long arm of the X-chromosome on the genloci Xq28. Over 217 Mutations have been characterized to date [9, 10]. Most of them are single base substitutions. The most common mutations described in Africa are A+, A− (202A) and Mediterranean. Non-synonymous mutations can cause different degrees of enzyme deficiency by activity or stability loss of the enzyme. As the gene is located on the X-chromosome, women are either homozygous deficient, heterozygous deficient (one gene encoding a normal enzyme and one gene encoding a deficient enzyme) or homozygous normal. The phenotype of heterozygous females varies between normal and deficient due to the X-chromosome inactivation in females expressing only one gene copy in each cell (mosaicism). Males are either hemizygous normal (normal phenotype) or hemizygous deficient (deficient phenotype) [11]. A prevalence of 0.5–2.9% of G6PD deficiency in the male hemizygote population was generally assumed for Ethiopia [12, 13]. However, a recent study in Southwestern Ethiopia reported higher prevalence of G6PD deficiency of up to 14.3% depending on the ethnic group of the patient [14]. A genetic study from Jimma detected A+ in 23.3% of the individuals and other uncommon mutations [15].

PQ is not part of the national Ethiopian policy as G6PD testing is not implemented at healthcare facility level. Population screening is recommended in regions with a G6PD deficiency prevalence ≥ 3–5% to guide decision-making for 8-aminoquinoline treatment in order to minimize the risk of drug-induced complications but population-based G6PD studies in Ethiopia are patchy [16].

This small study intended to collect first data on G6PD deficiency measured by spectrophotometry in combination with the analysis of associated genetic variants in healthy participants in Jimma zone. Test methods, procedures and logistics were additionally evaluated.

## Methods

### Recruitment and selection of samples

From March to June 2014, 206 participants without any life-threatening diseases and with unknown G6PD activity status were recruited on a voluntary basis at Jimma University, Ethiopia. Jimma is the capital of the Oromiya region and is situated 1780 m above sea level. Malaria is seasonal with higher prevalence from October to December (major peak) and from April to May (minor peak) [1].

The inclusion criteria for the G6PD study in Jimma were: age above 18 years, written informed consent, and permanent residency in the study area. The exclusion criteria involved acute or life-threatening illness, pregnancy and breastfeeding, simultaneous participation in another research study.

All data was reported and saved in specific questionnaires (Additional file 1) and transferred to an electronic data base via double entry. Questionnaires were regularly checked by the study coordinator and stored with restricted access. All study personnel had to sign a confidentiality agreement. Study results were communicated and explained to the participants and a G6PD card (Additional files 2, 3 and 4) with name and enzyme activity level was handed out. The study was approved by the ethical committee of the Jimma University and the Ludwig-Maximilians-University of Munich.

### Laboratory procedures

Venous blood was collected in 2.7 ml tubes containing ethylenediaminetetraacetic acid (EDTA), and following tests were performed within 6 h: the haemoglobin concentration in 15 μl blood was measured by rapid diagnostic test according to the manufacturer’s instructions (Haemocue AB, Ängelholm, Schweden). A thin (10 μl) and thick smear (10 μl) were prepared for microscopy. Rapid diagnostic tests for malaria (Binax NOW^®^ Malaria, Alere, USA) and G6PD enzyme activity (Binax NOW^®^ G6PD, Alere, USA) were performed according to the manufacturer’s instructions. Blood (6 × 15 µl) was dropped on filter papers (Whatman^®^ No 3, Sigma-Aldrich, Germany) for molecular investigations of the G6PD gene, air-dried, packed in plastic bags and stored at − 20 °C. During transport the filter papers were stored at room temperature for a maximum of 48 h.

#### Determination of G6PD activity via G6PD rapid diagnostic test, BinaxNOW, Alere, USA

In brief, 10 µl of EDTA blood and 70 µl of lysis reagent (red dyed buffer) were mixed in a microcentrifuge tube. Afterwards 50 µl of the mixture were placed in the middle of the reaction pad of the test device. After 7 min the result could be read through the viewing window. All procedures were done between 18 and 25 °C and protected from direct light. The results were read visually and were evaluated as positive/G6PD-deficient when the colour did not change and stayed light red. Negative/non-deficient samples changed the colour into a brownish dark colour on the upper part of the reaction pad.

#### Determination of G6PD-activity via spectrophotometry

An EDTA tube was stored at 4 °C until shipment to the University of Ulm, Germany. Samples were shipped via DHL or transported directly by study staff. Samples were stored at ambient temperature during transport and were immediately processed after arrival in Ulm. In Ulm, spectrophotometry for G6PD was performed by trained laboratory technicians. Haemoglobin was again measured during the process.

G6PD enzymatic activity in erythrocytes was measured by the method of Löhr and Waller [17]. A reaction mix of 1350 µl TRAP buffer (triethanolamine 50 mM, ethylenediaminetetraacetic acid 5 mM pH 7.5), 50 µl of magnesium chloride 1 M, 25 µl of haemolysate (0.9% w/v sodium chloride saline washed erythrocytes disrupted with a saturated solution of digitonine), 25 µl of NADP (nicotinamide-adenine-dinucleotide phosphate 30 mM) was started by finally adding 25 µl of G6P (glucose 6 phosphate, 50 mM). Photometric extinction rate compared to a blank without haemolysate was recorded at 37 °C. Results of the G6PD testing values were calculated in units per µMol metabolic rate/10^11^ erythrocytes/min (normal value: 30.5 ± 4.5). Activity was then converted into Units per g Haemoglobin (U/g Hb, Trinity Biotech for 37 °C at 366 nn, normal range 4.6–13.5 U/g Hb) by the following formula: (activity in μmol metabolic rate/10^11^ erythrocytes/min * RBC M/µL * 0.66)**/**Hb g/dL.

#### Genotyping

The genotyping of *G6PD* gene performed at the University of Ulm. Genomic DNA was purified using QIAamp DNA Blood Mini Kit from Qiagen according to the manufacturer’s instructions. Four PCR fragments were amplified containing exons 2, 3–5, 6–8 and 8–13, respectively, using specific primers (Table 1) and My Taq HS mix (Bioline^®^). The cycling conditions were the following: 10 cycles of heating at 98 °C for 15 s, 68° (with a decrease of 1 °C each cycle) for 1 min, 72 °C for 1 min 30 s. Followed by 25 cycles of heating at 98 °C for 15 s, 58 °C for 30 s and 72° for 1 min 30 s followed by an extension period of 10 min at 72 °C. PCR products were checked by agarose gel electrophoresis and purified with QIAquick PCR Purification Kit according to manufacturer’s instructions (Qiagen, Hilden, Germany).

**Table 1:** Primers used for sequencing

Name	Sequence 5′-3′		
G6PD_X153760116	TGTGCAGCTGAGGTCAATGG		ex13 R
G6PD_X153761532	ACTCGAGATGGACCAGGGTG	ex9 F	
G6PD_X153762859	ATGACCCTCTGGCTCAACAC		ex5 R
G6PD_X153764607	CGCAGGAGATGCGTTTATGTC	ex3 F	
G6PD_X153761674	GTGGTGACTTCTCCGGGGTT		ex8 R
G6PD_X153762867	GAGGGTCATCTGGGAACACA	ex6 F	
G6PD_12970-20-F	AGGTTCTGGCCTCTACTCCC	ex6 F	
G6PD_11852-20-R	GAGAAAACGCAGCAGAGCAC		ex4 R
G6PD_12051-20-F	GACGGGGACACTGACTTCTG	ex5 F	
G6PD_12478-20-R	CGCTCATAGAGTGGTGGGAG		ex5 R
G6PD_13631-20-R	AGGAGCTCCCCCAAGATAGG		ex7 R
G6PD_13773-20-F	GGAAGACAAGGGGGATCAGG	ex8 F	
G6PD_14079-18-R	GCATGCACACCCCAGCTC		ex8 R
G6PD_14624-20-F	GTGGGATGGTAGGTGATGCC	ex10 F	
G6PD_15515-20-R	TTCATCAGCTCGTCTGCCTC		ex12 R
G6PD_01253-20-F	CATCAACCACTCCCCAATGC	ex2 F	
G6PD_01677-20-R	GATCCTGGCGCACTAGCAG		ex2 R

For each PCR product the respective exons were sequenced with specific primers (Table 1) by a Beckman Coulter CEQ 8800 Analyzer (Beckman Coulter CEQ 8800, DTCS Kit). Sequences were compared to GenBank Accession Number X55448.1

#### Haemoglobinopathy screening

The thalassaemia screening was performed with hydragel electrophoresis with pH 8.6.

### Statistics

Questionnaire data and laboratory results were analysed by using Stata 14 (Statacorp, College Station, USA). Relevant continuous data were summarized by the following parameters: range, median and quartiles or mean and standard deviation if normally distributed. Categorical data are presented in contingency tables with frequencies and percentages of each value. The G6PD median activity value for normal males was calculated as previously described [18]. For the adjusted median activity, males with severe G6PD deficiency defined as ≤ 10% of the G6PD median value have to be excluded. Different cut-off levels of activity were defined according to previous recommendations (i.e., 10%, 30%, 70% and 80%). Individuals were classified as G6PD-deficient or normal based on these levels. Correlations were calculated by linear regression models.

The confidence level was 0.3–5.7%, if a prevalence of 3% in the male population was assumed and a sample size of 150 male participants and a confidence level of 95% were chosen. The power was 91% to detect between 1 and 6 cases if the prevalence was 3%. To compensate possible decay or other problems with some samples, the collection of 160 male samples was planned.

## Results

### Study population

Between March to and May 2014, 206 participants from the Oromiya region in Ethiopia were recruited (Table 2). Most of the participants originated from the Oromiya region (88.4%). 28.0% of them came from Jimma town directly, the others were comprised under Oromiya zone as they came originally from smaller towns and villages of the Oromiya region (Southwestern Ethiopia). Five per cent were born and raised in Addis Abeba (non-malaria endemic area). Other origins were Somali region, Harar, Amhara region (Bahir Dar) and Gondar (6.8%). All regions except for the capital are more or less malaria endemic with higher malaria prevalence in rural and lower areas. The age of the tested persons ranged between 18 and 45 years with a median of 22 years (IQR 21; 24). Only 20.4% of the participants were female.

**Table 2:** General information, n = 206

Gender	
Female, n (%)	42 (20.4)
Age in years, median (IQR)	22 (21; 24)
Origin, n (%)	
Oromiya zone (w/o Jimma Town)	131 (63.6)
Jimma Town	51 (24.8)
Addis Abeba	10 (4.8)
Other	14 (6.8)
Malaria positive, n (%)	0
Haemoglobin in g/dl, median (range)	16.8 (11.9–23.3)
Haemoglobin, male in g/dl, median (range)	17.1 (14.2–23.3)
Anaemia, male (< 14 g/dl), n (%)	0
High haemoglobin, male (> 18 g/dl), n (%)	38 (23.0)
Haemoglobin, female in g/dl, median (range)	14.3 (11.9–23.0)
Anaemia, female (< 12 g/dl), n (%)	1 (2.4)
High haemoglobin, female (> 16 g/dl), n (%)	5 (11.6)

None of the participants had fever or any relevant disease. Two subjects reported malaria in the medical history. No participant reported known G6PD deficiency or haemolytic crises in the medical history. Side effects after drug intake were reported for norfloxacin (1), ciprofloxacin (1), quinine (1), erythromycin (1), amoxicillin (2), cotrimoxazole (1), but could not further be specified.

### Laboratory findings

The median haemoglobin (Hb) for males and females was 17.1 (range 14.2–23.3) g/dl and 14.3 (11.9–23.0) g/dl, respectively (Table 2). There was only one slightly anaemic female in the whole population (11.9 g/dl). 23.0% of the males and 11.6% of the females showed elevated Hb levels. The mean Hb measured in Jimma directly by HemoCue (Haemocue AB, Ängelholm, Schweden) was 16.4 g/dl (95% CI 16.08–16.69) compared to 16.9 g/dl (95% CI 16.65–17.23) measured in Ulm. For further calculations, the Hb values measured in Ulm were used.

#### Malaria

No asymptomatic parasitaemia was detected by rapid test or microscopy.

#### Haemoglobinopathy

No thalassaemia or any known haemoglobinopathy were found.

#### Determination of G6PD activity

**Fig. 1:**
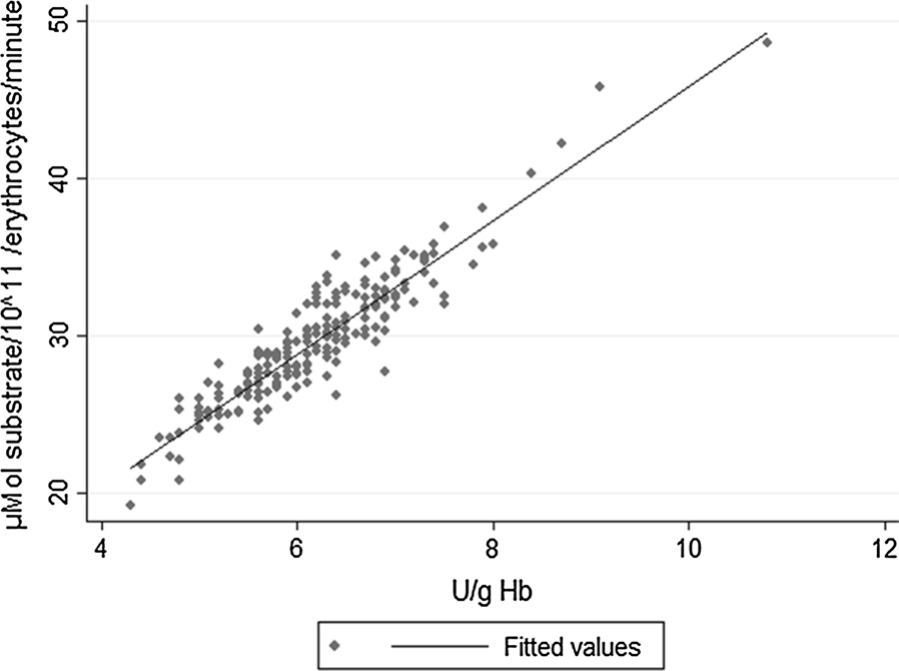
Correlation of G6PD activities in two different units. Correlation between G6PD activities given in µmol substrate conversion/10^11^ erythrocytes/min and U/g Hb (p < 0.000). The adjusted R^2^ was 0.8594

For the final analysis, 204 subjects were included (Table 3). Median G6PD activity in μmol metabolic rate/10^11^ erythrocytes/min was 29.1 (range 19.2–48.6) [normal value = 30.5 ± 4.5] (Fig. 1). The median activity was then 6.1 U/g Hb (range 4.3–10.8 U/g Hb). Two subjects showed very low enzyme activities (0.8 and 2.9 U/g Hb) and were excluded from the analysis because control enzyme activity [hexokinase, glucose-6-phosphate isomerase, triose-phosphate isomerase, pyruvate kinase 0.4, pyruvate kinase 2.0, glyceraldehyde-3-phosphate dehydrogenase, glutathione reductase, glutamic oxaloacetic transaminase (= aspartate transaminase)] was too low to yield reliable results for G6PD activity.

**Table 3:** Reference values for G6PD activity in the study population

G6PD activity in μmol metabolic rate/10^11^ erythrocytes/min^a^; in U/g Hb^b^	All (n = 204)	Male (n = 163)	Female (n = 41)
Median	29.1; 6.1	29.1; 6.1	29.6; 6.4
IQR	(26.9; 32.0); (5.6; 6.7)	(26.9; 32.3); (5.6; 6.6)	(27.2; 31.9); (6.0; 6.9)
Range	19.2–48.6; 4.3–10.8	19.2–48.6; 4.3–10.8	20.8–42.2; 4.4–8.7
Mean	29.59; 6.17	29.55; 6.11	29.69; 6.40
SD	3.99; 0.87	3.96; 0.85	4.12; 0.93

^a^Normal value = 30.5 ± 4.5

^b^Normal value 4.6 − 13.5

**Fig. 2:**
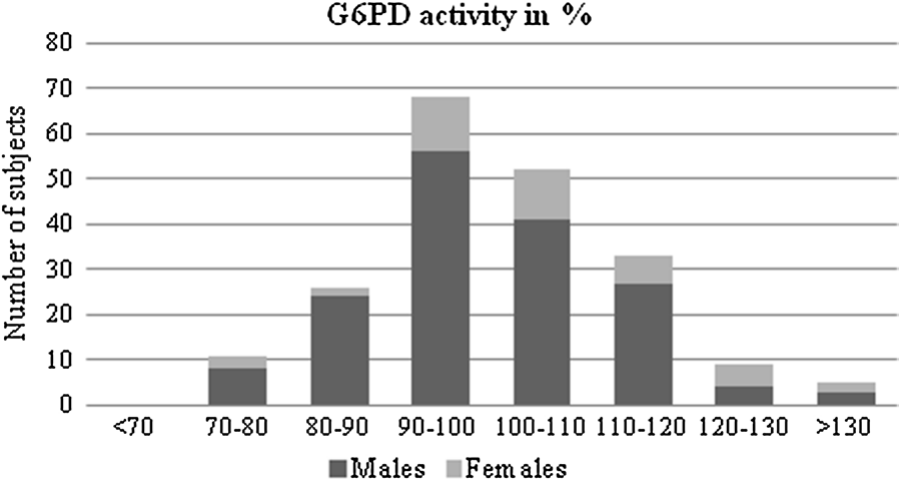
G6PD activity in per cent. The graph shows the distribution of G6PD activity in per cent for the study population, two very low values were assumed invalid as control enzyme activity was also very low, they were not included in the analysis (n = 204). Normal activity was defined as the median activity in U/g Hb in the male population and set 100%. No values below 70% were detected

To determine the normal activity for this population, the results of all males (n = 164) were used. An adjustment as previously described to avoid having severely deficient values skew the population median was unnecessary [19]. The distribution was unskewed (Fig. 2). The male median was 6.1 (4.3–10.8) and set 100% of the activity in the study population. The female median was 6.4 (range 4.4–8.7).

A total of three participants presented lower values than the defined normal range (< 4.6 U/mg Hb) but there was no enzyme activity below 10% (severe) or 60% (moderate deficiency) [12] (Table 4, Fig. 1). Lowest value was 4.3 U/g Hb correlating to 70.5% enzyme activity. There were 11 participants with values between 70 and 80% enzyme activity (Fig. 2, Additional file 5: Table S1), three of them were female.

**Table 4:** G6PD genotypes and associated G6PD activity values of 34 sequenced samples

G6PD activity in μmol substrate/10^11^ erythrocytes/min^a^	G6PD activity in U/g Hb^b^	G6PD activity in %^d^	Genotype	Gender	Origin (malaria endemic ±)
*3.9* ^c^	*0.8* ^c^	13.1^c^	Normal	M	Harar+
*13.9* ^c^	*2.9* ^c^	47.5^c^	Normal	F	Addis−
*19.2*	*4.3*	70.5	G6PD-A(+)	M	Oromiya+
*21.8*	*4.4*	72.1	Normal	F	Jimma+
*20.8*	*4.4*	72.1	Normal	F	Oromiya+
*23.5*	4.6	75.4	G6PD-Jimma	M	Jimma+
*23.5*	4.7	77.1	Normal	F	Jimma+
*22.3*	4.7	77.1	Normal	M	Somali+
*22.1*	4.8	78.7	Normal	M	Oromiya+
*20.8*	4.8	78.7	Normal	M	Oromiya+
*23.8*	4.8	78.7	Normal	M	Oromiya+
*24.1*	4.9	80.0	Normal	M	Jimma+
*24.6*	4.9	80.0	Normal	M	Oromiya+
*25.1*	5.0	82.0	Normal	M	Jimma+
*24.8*	5.1	83.6	Normal	M	Oromiya+
*24.1*	5.2	85.2	Normal	M	Jimma+
*24.9*	5.2	85.2	Normal	M	Oromiya+
*25.0*	5.3	86.7	Normal	M	Jimma+
*25.1*	5.4	88.5	Normal	M	Oromiya+
26.5	5.4	88.5	Normal	M	Oromiya+
27.5	5.5	90.2	Normal	M	Jimma+
*24.6*	5.6	91.8	Normal	M	Somali+
27.3	5.6	91.8	Normal	M	Jimma+
26.1	5.9	96.7	Normal	M	Oromiya+
26.7	6.0	98.4	Normal	M	Oromiya+
28.1	6.1	100.0	Normal	F	Jimma+
30.0	6.3	103.3	Normal	M	Jimma+
30.8	6.4	104.9	Normal	M	Jimma+
29.0	6.4	104.9	Normal	M	Jimma+
30.4	6.7	109.8	Normal	F	Jimma+
33.0	6.8	111.5	Normal	M	Jimma+
27.7	6.9	113.1	Normal	F	Jimma+
35.4	7.1	116.4	Normal	M	Jimma+
35.6	7.9	129.5	Normal	F	Bahir Dar+

^a^In bold values below 26.0

^b^In bold values below 4.6

^c^Invalid values, G6PD activity values excluded from the analysis

^d^U/g Hb as reference

There was no difference between the gender and spectrophotometry results measured in μmol metabolic rate/10^11^ erythrocytes/min (p = 0.7922). Measured in U/g Hb, women (6.4 U/g Hb) had significantly higher values of enzyme activity than men (6.1 U/g Hb; p = 0.0315).

Mean storage time (time between blood sampling and spectrophotometry measurement) was 12.2 days (SD 5.2, range 4–21 days). There was no correlation between storage time (time between blood sampling and spectrophotometry measurement) and spectrophotometry results (adjusted R^2^ = 0.01; Table 5).

**Table 5:** Storage time and G6PD activity in U/g Hb, n = 204

Time in days from blood drawing to spectrophotometry	n (%)	Median G6PD activity in U/g Hb [IQR]
4	7 (3.4)	5.9 [5.0; 6.3]
5	12 (5.9)	6.2 [5.6; 6.8]
6	29 (14.2)	5.9 [5.3; 6.3]
7	24 (11.8)	6.4 [6.1; 6.8]
10	12 (5.9)	5.4 [4.5; 6.3]
11	10 (4.9)	6.1 [5.6; 6.6]
12	2 (1.0)	6.2 [5.5; 6.8]
13	7 (3.4)	5.9 [5.5; 6.7]
14	28 (13.7)	6.5 [6.1; 7.0]
16	23 (11.3)	6.4 [5.7; 7.4]
17	12 (5.9)	6.1 [5.7; 6.4]
18	12 (5.9)	6.1 [5.5; 6.4]
19	15 (7.3)	6.1 [5.5; 7.0]
21	11 (5.4)	6.3 [5.6; 6.9]

#### G6PD rapid test

The RDT showed negative (= normal) results for all samples. The false positive rate was 0%. There was no discrimination between lower and higher values in this study group. All samples with slightly diminished but normal enzyme activity (70–80%) in spectrophotometry indicated normal activity by RDT. The two control samples from the University of Ulm with severe deficiency (< 10% enzyme activity) were correctly discriminated.

#### Genotyping

The sequencing of the *G6PD* gene was processed for 34 out of 206 samples based on low measured activity in spectrophotometry, gender and origin (Table 4). Genotyping of these samples showed one G6PD-A(+) variant at position 376 (no enzyme defect) and one mutation at position 445 (G->A) resulting in an amino acid substitution (A149T) not described before (Table 6).

**Table 6:** Mutations of the g6pd gene found in the study population

Mutation	Base substitution	Position	Amino acid substitution	Zygosity	Gender	Origin
G6PD-A(+)	A → G	376	N126D	Hemizygous	M	Oromiya
G6PD-Jimma	G → A	445	A149T	Hemizygous	M	Jimma

## Discussion

The median activity in the study population was 6.1 U/g Hb (range 4.3–10.8 U/g Hb). No activity below 70% or 4.27 U/g Hb could be detected. A prevalence of 0.5–2.9% of G6PD deficiency in the male hemizygous population is generally assumed for Ethiopia. In comparison, prevalence of G6PD deficiency in the male population in other African countries are reported as follows: Somalia: 0.5–2.9%, Sudan: 7.0–9.9%, Uganda, Kenya: 10.0–14.9%, Tanzania, Congo, Gabon and Cameroon: 15.0–26.0% [12, 13]. As Tsegaye et al. published before, the prevalence of G6PD deficiency in Gambella, Ethiopia, varied between ethnic groups. Overall prevalence of G6PD deficiency measured by CareStart™ G6PDd screening test (Access Bio, Inc., New Jersey, USA) was 7.3%. Gambella is low land area (300–500 m above sea level) characterized by high malaria transmission. They reported high prevalence among the indigenous group of Nuer (14.3%) and Anuak (12.0%) in rural areas and no deficiency in 46 patients from Oromiya [14]. This corresponds to the 0% prevalence in the presented study. Patients from other “highland” regions like Amhara, Hadya and Tigre showed also no deficiencies [14]. According to genetic studies, the Oromo and Amhara people appear more similar to Europoids (particularly to the South Arabians) and quite different from the Negritic people. Intermixture of Europoid and Negritic populations seem to have resulted in the present day Cushitic (and Semitic)-speaking group of eastern Africa [20].

As there are no other studies published from Ethiopia measuring the G6PD activity by spectrophotometry, a local comparison of the median level in U/g Hb had to be omitted. A study from the US examining participants with Afro-American origin reported a median G6PD activity of 7.30 U/g Hb. Male median was unadjusted 7.06 and adjusted 7.18, female median was 7.70 U/g Hb [19]. The female median was higher than the male. This is in accordance to the results here. Measured in U/g Hb, women (6.4 U/g Hb) had significantly higher values of enzyme activity than men (6.1 U/g Hb; p = 0.0315) as the haemoglobin value differed significantly (p < 0.0001) between the sexes, with women having lower Hb levels than men. There was no difference between the sexes when the enzyme activity was expressed in μmol metabolic rate/10^11^ erythrocytes/min.

In another study from Bangladesh with 999 participants, the adjusted male median derived by spectrophotometry was 7.03 U/g Hb [21]. Compared to the two studies above, the overall median in the presented study was lower. Several reasons are possible. The conversion formula used is an approximation only. Furthermore, G6PD testing in the field poses many challenges to the study team. Loss of enzyme activity during the process cannot be excluded. As Ley and colleagues point out, the duration between blood collection and spectrophotometry and the storage conditions in between are the crucial factors to maintain a stable enzyme activity [7].

G6PD enzyme activity and degradation are temperature dependent, EDTA blood should be cooled at least at 4–8 °C. The possible duration without loss of activity is unknown [7]. One study reports a loss of a maximum of 5% of activity after 21 days storage at 4 °C [22]. However, others report loss of activity of 15–21% after 21 days, 6% after 7 days and 40% after 7 days at 4 °C [7]. Cryopreservants and storage at − 80 °C [23] or replacing plasma with an additive [22] are recommended.

The samples of this study were constantly cooled at 4 °C until shipment. Transport via DHL express (4–7 days) or by study members (2 days) was at ambient temperature. Shipment via DHL was problematic due to logistical problems and customs issues and cannot be safely recommended. Overall mean storage time was 12.2 days (SD 5.2, range 4–21 days). Storage duration and G6PD activity levels showed no evident correlation to indicate a systematic significant loss of activity over time. Haemolysis and significant loss of enzyme activity was only suspected in two samples with overall low activities for nine measured control enzymes and a storage time of 14 and 19 days. Median G6PD activity in samples stored 14 and 19 days was 6.5 and 6.1 U/g Hb, respectively.

Assuming a normal male median of 7.0 U/g Hb [19, 21] a median of 6.1 U/g Hb would mean a loss of 13% in enzyme activity. The real absolute values in the study could be slightly higher than the presented values. On the other hand, the haemoglobin level was relatively high with 17.1 g/dl for males and 14.3 g/dl for females. Twenty percent of the study population had elevated haemoglobin levels. All participants, though originating from different villages, were living in Jimma town that is situated at 1780 m above sea level. Higher haemoglobin levels result in lower G6PD values as the calculation with the formula used is haemoglobin-dependent. The two studies cited above presented no haemoglobin levels for comparison but were conducted with participants living at sea level [19, 21]. In conclusion, activity loss cannot be excluded but relevant loss seems unlikely; relative values in per cent should not be affected in any way.

Genotyping of the *G6PD* gene revealed two mutations in the small sub-population. The G6PD-A+ mutation (A376G) was found in one male (2.9%). Enzyme activity was 4.3 U/g Hb (70.5%). He reported an intolerance to norfloxacin that was not further specified but no haemolysis was reported in the medical history. G6PD-A−, the combination of A376G and G202A, and C563T (G6PD-Mediterranian) are common variants in Africa, the Mediterranean region and the Middle East but G202A and C563T could not be found in this study. This is in accordance with another study from Southwestern Ethiopia, Malo, genotyping 555 samples for G202A and C563T. None of the variants were detected [24]. A recent paper reported G6PD genetic variants in malaria patients from Jimma zone [15]. They found G6PD-A+ in 23.3% of the 86 individuals whereas G202A and C563T were absent. They reported other mutations: G535A in two females and two additional mutations in the intronic region in one male (485 + 37 G → T, rs370658483, chrX: 154535131).

While G6PD-A+ is usually regarded as variant with normal enzyme function, G6PD-A− was associated with a milder degree of enzyme deficiency. However, recent reports suggest that G6PD-A− was associated with severe illness after administration of primaquine [25, 26]. Primaquine sensitivity has only been reliably characterized for the variants Mediterranean and Mahidol with severe and moderate degrees of sensitivity to primaquine, respectively [27]. The second mutation that was observed in this study was the substitution G445A. The male participant showed an enzyme activity of 75.4%. He originated directly from Jimma town. This variant was not observed before.

Considering the results presented, would there be any consequences concerning radical cure of *P. vivax* for the subjects of the study? A generally adopted method and its corresponding cut-off for G6PD deficiency would simplify comparison and implementation of standards. The recommended thresholds concerning malaria treatment are indicated in percent. PQ treatment for radical cure in vivax malaria (0.25–0.5 mg/kg/day for 14 days) should not be given in individuals with G6PD activity below 30% of the adjusted male median (AMM). This level excludes severe deficiency and seems safe to discriminate homozygous females and hemizygous men from heterozygous females or variants causing only moderate deficiency [27]. For the long-acting tafenoquine, the recommended threshold in the approval studies was 70% of the AMM for all participants and 90% for anaemic females (7–10 mg/dL) [28] to exclude moderate deficiencies.

According to the WHO, heterozygote females are classified in activity levels of < 30%, 30–80% and > 80% correlating in a deficient, intermediate or normal phenotype, respectively. Sensitivity to primaquine seems possible in heterozygote females with the intermediate phenotype and unlikely in heterozygote females with the normal phenotype [29]. Eleven patients showed activities between 70 and 80% of the male median in this study: three females and eight men with the normal genetic variant and two hemizygote men with the stated mutations above. Both showed a borderline intermediate phenotype. Sensitivity to 8-aminoquinolines seems at least possible.

If the above-mentioned thresholds of 30% for PQ and 70% for tafenoquine are used, both regimens could be administered to the study population. However, the males with the G6PD variants should be treated with caution and closely monitored. There is few or no information about 8-aminoquinoline sensitivity concerning these mutations.

From a clinician’s perspective, a cut-off of 80% would prevent any possible, probably mild harm in two cases (1.0%) but nine healthy patients (4.4%) would miss the valuable radical cure. From a public health point of view, this approach lacks efficiency and a lower cut-off should be considered, but a general recommendation to treat without testing in this population would be irresponsible as the study population is not representative enough to safely exclude severe deficiency. However, in absence of a better drug the low prevalence of G6PD deficiency should encourage further malaria elimination efforts in this area, as the chances of an effective and broadly applicable use of 8-aminoquinolines are high.

No parasitaemia was found by RDT or microscopy in this study. Recruitment was between March and May during and after the smaller rainy season. A recent study from rural Southwestern Ethiopia conducted in a similar mountainous region with seasonal malaria found asymptomatic sub-microscopic parasitaemia with *P. falciparum* in 5.2%, *P. vivax* in 4.3% and mixed infection in 0.2% of 555 individuals by PCR [24]. Microscopy and RDT were negative in all samples. Therefore, sub-microscopic parasitaemia cannot be excluded in the underlying study group although probability seems lower as all participants lived in Jimma town in contrast to rural areas. Microscopy and RDT (Binax NOW^®^ Malaria, Alere, USA) correlated well so far.

Evaluation of the G6PD RDT (Binax NOW^®^ G6PD, Alere, USA) was impossible due to the small sample size in combination with zero prevalence of deficiency. Discrimination of slightly diminished enzyme activity (70–80%) and normal activity was impossible as expected.

## Conclusion

The low prevalence of G6PD deficiency was unsurprising as individuals originating mainly from a highland area of Ethiopia were investigated. Though, genetic variants with enzyme activity between 70 and 80% could be detected. The zero prevalence of severe deficiency might not encourage population-based screening studies or routine screening projects of newborns for G6PD deficiency. However, routine screening for G6PD deficiency during a malaria infection should be implemented in vivax endemic areas to enable the administration of the relatively cheap radical cure regimen that would enormously booster elimination efforts. Administration of 8-aminoquinolines in G6PD-A− and G6PD-A+ individuals and others with common variants should be further investigated to safely determine a feasible and safe cut-off. At the moment, the cut-off of 70% for tafenoquine is only safely measurable by spectrophotometry. The G6PD RDT was unable to discriminate at this cut-off and seems only valuable for excluding severe deficiency.

The WHO recommends incorporation of G6PD testing into national guidelines and available services possibly with referral of patients from lower to higher level health facilities where both G6PD testing and radical cure can be provided [29]. As a first step, spectrophotometry could be introduced at Jimma University and other high-level healthcare facilities and radical cure offered in their out-patient departments and hospitals. A referral system could be developed in the future. Health facilities in rural areas could benefit from new field-approved alternatives that have to be enhanced and evaluated. Cheap discrimination tests to exclude severe deficiency could be an interim solution as other drugs than 8-aminoquinolines can cause haemolytic crises in case of deficiency.

In this study, participants received a G6PD card explaining their individual enzyme activity. The attractiveness of genetic tests is based on the fact that a patient once tested and familiar with the test result has not to be tested twice.

## Additional files

**Additional file 1.** Questionnaire.

**Additional file 2.** G6PD Card (Englisch).

**Additional file 3.** G6PD Card (Oromo).

**Additional file 4.** G6PD Card (Amhara).

**Additional file 5: Table S1.** G6PD activity in per cent and associated gender distribution.
