# Supplementary material for: Glucose-6-phosphate dehydrogenase activity measured by spectrophotometry and associated genetic variants from the Oromiya zone, Ethiopia
Source: Malar J. 2018 Oct 12;17:358. doi: 10.1186/s12936-018-2510-3 (PMC6186078; doi:10.1186/s12936-018-2510-3)
Supplement: Supplementary file 1 — Additional file 1. Questionnaire. [file 12936_2018_2510_MOESM1_ESM.pdf]

Day 0 (Recruitment)

Health Centre:______________________________________ Date:____/____/_________

Patient:_______________________________________ ID No: ________________________

Address/mobile number:____

Did the subject/or subject's representative sign the inform consent form?

□ Yes □ **No → Do not proceed!**

Age:__________(years) **!!! ≥ 4 years !!!**

Sex: □ male □ female

Origin: □ city □ rural

Temperature (°C):______________ Weigth (kg):____________

Medical history:

Any symptoms	□ No	If yes, please specify modality	Onset of symptoms
Fever		(°C) **(Exclusion criteria!)**	
Shivering		□ mild □ moderate □ severe	
Headache		□ mild □ moderate □ severe	
Nausea		□ mild □ moderate □ severe	
Diarrhoea		□ mild □ moderate □ severe	
Abdominal pain		□ mild □ moderate □ severe	
Others (please specify)		□ mild □ moderate □ severe	

Any symptoms for malaria? □ **Yes (→ Exclusion)** □ No

If yes, immediate referral to health centre or Jimma university hospital.

Is the female subject pregnant or breastfeeding? □ Yes □ No

Does the patient have any known illnesses? □ Yes □ No

If yes please mention in subject's history above.

Does the subject have any allergies? □ Yes □ No

If yes please mention in subject's history above.

Does the subject remember an episode of haemolysis? □ Yes □ No

If yes please mention in subject's history above.

Does the subject remember side effects after drug intake? □ Yes □ No
Which drugs?____

Did the subject ever receive blood transfusion? □ Yes □ No
Why?____

Does the subject currently take any medicine regularly? □ Yes □ No
If yes please mention:____(Why? Check medical history)

Laboratory Work

EDTA blood taken □ Yes

Next appointment

Date___/___/_______ Time: ___/___

______________________________ ____

Date, Place Researcher's Name, Signature

Thank you!

Laboratory Results Day 0

Health Centre:______________________________________ Date:____/____/_________

ID No: ________________________

Haemoglobin measured? □ Yes

Haemoglobin Results (mg/dl) □ Normal □ **Abnormal (see age scale)**

→Iron supplementation

Filter papers prepared (6 x 15 µL)? □ Yes

Thin and thick smear prepared? □ Yes

Rapid Test G6PD-Enzyme Activity: □ Normal □ **Deficient**

Rapid Test Malaria: □ negative □ **positive** if positive, species:_______________________________

Filter papers packed and frozen? □ Yes

Thin and thick smear:

□ negative □ positive (/µL:_____________________ Species:_______________________________

Gametocytes seen? □ No □ Yes (/µL:______________)

If malaria positive:

Patient informed and referred for treatment? □ Yes □ No

_________________________________ ____

Date, Place Researcher's Name, Signature

Thank you!

Visit 2 (Follow-up)

Health Centre:_______________________________________ Date:____/____/_________

Patient:____ ID No: _________________________

Address:_______________________________________

Had the subject a positive malaria test at day 0? □ **No** □ Yes

If yes (→ Proceed with CRF "Malaria patient follow-up")

G6PD-Enzyme Activity:	□	Normal	□	**Deficient**
G6PD Card given	□	**Yes**	□	No
G6PD Information given	□	**Yes**	□	No

__________________________________ ____

Date, Place Researcher's Name, Signature

Thank you!

Malaria Patient Follow-up

Health Centre:_____________________________________ Date:____/____/_________

Patient:______________________________________ ID No: ________________________

Had the subject a positive malaria test at day 0? □ No □ **Yes**

Rapid Test Malaria

□ negative □□ positive if positive, species:________________________________

Thin and thick smear:

□ negative □□ positive (/µL:_____________________ Species:______________________________

Patient already treated against malaria? □ **Yes** □ No

Drug:____

If no, referral to health centre or Jimma university hospital for malaria treatment!

Patient history since day 0:

Symptoms since day 0	NO	If yes, please specify modality	Onset/end of symptoms	Causality (1=related, 2=probable 3=possible, 4=unrelated)
Fever		(°C)		□ 1 □ 2 □ 3 □ 4
Shivering		□ mild □ moderate □ severe		□ 1 □ 2 □ 3 □ 4
Headache		□ mild □ moderate □ severe		□ 1 □ 2 □ 3 □ 4
Nausea		□ mild □ moderate □ severe		□ 1 □ 2 □ 3 □ 4
Diarrhea		□ mild □ moderate □ severe		□ 1 □ 2 □ 3 □ 4
Abdominal pain		□ mild □ moderate □ severe		□ 1 □ 2 □ 3 □ 4
Others (specify)		□ mild □ moderate □ severe		□ 1 □ 2 □ 3 □ 4

Any symptoms for malaria or other acute diseases now? □ Yes □ No

If yes, immediate referral to health centre or Jimma university hospital!

Page 2 malaria patient follow-up

G6PD-Enzyme Activity:	□	Normal	□	**Deficient**
G6PD Card given	□	**Yes**	□	No
G6PD Information given	□	**Yes**	□	No
Indication for primaquine?	□	Yes*	□	No

**Plasmodium vivax* or *P. ovale* infection

Contraindication for primaquine	□	No	□	**Yes (→ No primaquine!!!)**[§]

[§]Pregnancy, lactation, age < 4 years, G6PD deficiency, known allergy against primaquine

Treatment

Primaquine given?	□	Yes	□	No

(0.5 mg/kg KG once daily for 14 days given)

This treatment has to be combined with the standard chloroquine therapy.

________________________________ ____

Date, Place Researcher’s Name, Signature

Thank you!

Drop Out Form

Health Centre:______________________________________ Date:____/____/_________

Patient:_______________________________________ ID No: ________________________

- Screening Failure

  Reason:____

- Lost to Follow-up:

  Explanation:-____

- Withdrawal:

  Reason:-____

- Protocol violation:

  Explanation:-____

_________________________________ ____

Date, Place Researcher’s Name, Signature

Thank you!
