# Supplementary material for: Glucose-6-phosphate dehydrogenase activity measured by spectrophotometry and associated genetic variants from the Oromiya zone, Ethiopia
Source: Malar J. 2018 Oct 12;17:358. doi: 10.1186/s12936-018-2510-3 (PMC6186078; doi:10.1186/s12936-018-2510-3)
Supplement: Supplementary file 2 — Additional file 2. G6PD Card (Englisch). [file 12936_2018_2510_MOESM2_ESM.pdf]

Glucose-6-phospatdehydrogenase (G6PD) Enzyme Deficiency Card

Name:____

Birth date:_______________________________________

Address:____

The above named person has a G6PD enzyme deficiency of __________%.

This means a moderate/severe enzyme deficiency, most probably with an African variant. It was tested at Jimma University Hospital on _________________.

G6PD enzyme deficiency is an inherited disease of the red blood cell. It is not infectious but can be dangerous for the patient if certain drugs are administered. Those drugs can cause life-threatening haemolyses (destruction of red blood cells). Depending on the gene mutation (African, Asian, Mediterranean variant), some of the drugs are more or less dangerous.

The following drugs should not be given (high risk):

Acetanilide (acetanilid)	$C_8 H_9 N O$
Acetylphenylhydrazine (2-Phynylacetohydrazide)	$C_8 H_{10} N_2 O$
Aldesulfone sodium (sulfoxone)	$C_{14} H_{14} N_2 Na_2 O_6 S_3$
Arsine	As-H3
Beta-Naphthol (2-Naphthol)	C10 H8 O
Chloramphenicol	C11 H12 C12 N2 O5
Chloroquine	C18 H26 Cl N3
Ciprofloxacin	C17 H18 F N3 O3
Dapsone (diaphenylsulfone)	C12 H12 N2 O2 S
Dimercaprol	C3 H8 O S2
Doxorubicin	C27 H29 N O11
Furazolidone	C8 H7 N3 O5
Glibenclamide	C32 H28 Cl N3 O5 S
Glucosulfone (glucosulfone sodium)	C24 H34 N2 Na2 O18 S3
Isobutyl Nitrite	C4 H9 N O2
Menadiol Sodium Sulfate (Vitamin k4 sodium sulfate)	C11 H8 Na2 O8 S2
Menadione (menaphtone)	C11 H8 O2
Menadione sodium Bisulfite (Vitamin K3 sodium bisulfite)	C11 H8 O2 NaHSO3
Mepacrine (Quinacrine)	C23 H3O Cl N3 O
Mesalazine - 5-Aminosalicylic Acid (paraminosalicylic acid)	C7 H7 N O3
metamizole	C13H16N3NaO4S
Methyltioninium Chloride (methylene blue)	C16 H18 Cl N3 S
Nalidixic Acid	C12 H12 N2 O3
Naphtalene, Pure (naphtalin)	C10 H8
Niridazole	C6 H6 N4 O3 S
Nitrofural (nitrofurazone)	C6 H6 N4 O4
Nitrofurantoin	C8 H6 N4 O5
O-Acetylsalicylic Acid (acetylsalicylic acid)	C9 H8 O4

Oxidase, Urate (urate oxidase)	
Pamaquine	C42 H45 N3 O7
Pentaquine	C18 H27 N3 O
Phenacetin (acetophenetidin)	C10 H13 N O2
Phenazopyridine	C11 H11 N5
Phynylhydrazine	C6 H8 N2
Primaquine	C15 H21 N3 O
Probenecid	C13 H19 NO4 S
Stibophen (2-(2-Oxido-3,5-Disulphonatophenoxy)-1,3,2,Benzodioxastibole-4-6-Disulphonate)	C12 H4 Na5 O16 S4 Sb
Sulfacetamide	C8 H10 N2 O3 S
Sulfadimidine	C12 H14 N4 O2 S
Sulfafurazole (sulfafurazone, sulfisoxazole)	C11 H13 N3 O3 S
Sulfamethoxazole	C10 H11 N3 O3 S
Sulfanilamide (Sulphanilamide)	C6 H8 N2 O2 S
Sulfapyridine	C11 H11 N3 O2 S
Sulfasalazine, Salazosulfapyridine (salazopyrin)	C18 H14 N4 O5 S
Thiazosulfone (thiazolesulfone)	C9 H9 N3 O2 S2
Tolonium Chloride, Tolonium Chloride (toluidine blue)	C15 H16 Cl N3 S
Trinitrotoluene (2,4,6-Trinitrotoluene)	C7 H5 N3 O6

Following drugs are low risk drugs:

- Acetaminophen (**paracetamol**, Tylenol, Tralgon)
- Acetophenetidin (phenacetin)
- Aminopyrine (Pyramidon, amidopyrine)
- Antazoline (Antistine)
- Antipyrine
- Ascorbic acid (vitamin C)
- Benzhexol (Artane)
- Chloramphenicol [high risk for Mediterranean/Asian variants]
- Chlorguanidine (Proguanil, Paludrine)
- Chloroquine
- Colchicine
- Diphenhydramine (Benadryl)
- Isoniazid
- L-Dopa
- Menadione sodium bisulfite (Hykinone)
- Menapthone
- *p*-Aminobenzoic acid
- Phenylbutazone
- Phenytoin
- Probenecid (Benemid)
- Procain amide hydrochlonde (Pronestyl)
- Pyrimethamine (Daraprim)
- Quinidine
- Quinine
- Streptomycin
- Sulfacytine
- Sulfadiazine
- Sulfaguanidine
- Sulfamerazine
- Sulfamethoxypyridazine (Kynex)
- Sulfisoxazole (Gantrisin)
- Trimethoprim
- Tripelennamine (pyribenzamine)
- Vitamin K
