# Supplementary material for: Glucose-6-phosphate dehydrogenase activity measured by spectrophotometry and associated genetic variants from the Oromiya zone, Ethiopia
Source: Malar J. 2018 Oct 12;17:358. doi: 10.1186/s12936-018-2510-3 (PMC6186078; doi:10.1186/s12936-018-2510-3)
Supplement: Supplementary file 3 — Additional file 3. G6PD Card (Oromo). [file 12936_2018_2510_MOESM3_ESM.pdf]

Kaardii hirrina inzaayimii Giluukoos-6-fosfeeti diihayidiroojineesii (G-6-FD)

Maqaa: _______________________________.

Guyyaa dhalootaa: ______________________________.

Teessoo: _______________________________________.

Namichi/ttiin maqaan isaa/shee kanaa olitti caqasame/te kun hirrina G-6-FD ____________% qaba/di. Kana jechuunis hirrina giddugaleessa ykn cimaa inzaayimichaa, garaa-garummaa Afiriikummaa ta'uu ni mala. Qorannoon kun hospitaala yuniversiitii Jimmaatti guyyaa ______________________ hojjetame.

Hirrinnni inzaayimii G-6-FD kun gosaan dhalootaa gara dhalootaatti kan darbu ta'ee dhukkuba

seelii dhiiga diimaa ti. Dhukkuba ilbiisota ijaan hin argamneen kan daddarbu utuu hin ta'in dhukkubsattoonni yeroo qorichaalee tokko tokko fudhatanu kan cimuu danda'uudha. Korichootni kun seelii dhiiga diimaa barbadeessuudhaan hanga lubbuu baasuutti deemuu danda'u. Addaddummaa idda dhalootaatiin (Afiriikaa, Eeziyaa, Meditaraaniyaan, kkf) qorichootiin murtaa'ani miidhaa cimaa ykn laafaa ta'uu ni danda'u.

Odeeffannoon dhukkuba kanaa qorannoo inzaayimii godhatanii ji'a 3n booda hospitaala Yunivarsiitii Jimmaatti Dr. Sintaayyoo Fiqaaduutiin kennamuu ni danda'a.

Qorichootni kanaan gaditti caqasaman rakkina cimaa waan huumaniif kennamuu hin qabanu.

Asitaaniilamaayid (asitaaniliid)	C8 H9 N O
Asetiilfeniilhayidiraziin(2-Feniilasetohayidraazaayid)	C8 H10 N2 O
Aldeesalfoon soodiyeem (salfoksoon)	C14 H14 N2 Na2 O6 S3
Arsiin	As-H3
Beettaa-naaftool (2-naaftool)	C10 H8O
Kilooraamfenikool	C11 H12 C12 N2 O5
Kilorokiin	C18 H26 Cl N3
Sippiroofiloksaasiin	C17 H18 F N3 O3
Dapsoon (diyafenylsalfoon)	C12 H12 N2 O2 S
Dayimerkappirool	C3 H8 O S2
Doksoruubisiin	C27 H29 N O11
Fuuraazoliidoon	C8 H7 N3 O5
Gilibenkilaamaayid	C32 H28 Cl N3 O5 S

Gilukosalfoon (gilukosalfoon soodiyeem)	C24 H34 N2 Na2 O18 S3
Ayisobutiilnaayitiraayit	C4 H9 N O2
Menadiyol sodiyeem salfeet (vaayitamin k4 soodiyeem salfeet)	C11 H8 Na2 O8 S2
Meppaakiriin (kuyinaakiriin)	C11 H8 O2
Meesaalaaziin-5-amiinoosalisiliik asiid (paaraamiinoosalisiliik asiid)	C7 H7 N O3
Metaamizool	C13H16N3NaO4S
Meetiiltiyooniyeem kilooraayid (meetiliin biluu)	C16 H18 Cl N3 S
Nalidiksiik asiid	C12 H12 N2 O3
Naftaaliin	C10 H8
Niridaazool	C6 H6 N4 O3 S
Nayitiroofural (nayitiroofuraazoon)	C6 H6 N4 O4
Nitrofurantoin	C8 H6 N4 O5
O-Acetylsalicylic Acid (acetylsalicylic acid)	C9 H8 O4
Oxidase, Urate (urate oxidase)	
Pamaquine	C42 H45 N3 O7
Pentaquine	C18 H27 N3 O
Phenacetin (acetophenetidin)	C10 H13 N O2
Phenazopyridine	C11 H11 N5
Phynylhydrazine	C6 H8 N2
Primaquine	C15 H21 N3 O
Probenecid	C13 H19 NO4 S
Stibophen (2-(2-Oxido-3,5-Disulphonatophenoxy)-1,3,2,Benzodioxastibole-4-6-Disulphonate)	C12 H4 Na5 O16 S4 Sb
Sulfacetamide	C8 H10 N2 O3 S
Sulfadimidine	C12 H14 N4 O2 S
Sulfafurazole (sulfafurazone, sulfisoxazole)	C11 H13 N3 O3 S
Sulfamethoxazole	C10 H11 N3 O3 S
Sulfanilamide (Sulphanilamide)	C6 H8 N2 O2 S

Sulfapyridine	C11 H11 N3 O2 S
Sulfasalazine, Salazosulfapyridine (salazopyrin)	C18 H14 N4 O5 S
Thiazosulfone (thiazolesulfone)	C9 H9 N3 O2 S2
Tolonium Chloride, Tolonium Chloride (toluidine blue)	C15 H16 Cl N3 S
Trinitrotoluene (2,4,6-Trinitrotoluene)	C7 H5 N3 O6

- Acetaminophen (paracetamol, Tylenol, Tralgon)
- Acetophenetidin (phenacetin)
- Aminopyrine (Pyramidon, amidopyrine)
- Antazoline (Antistine)
- Antipyrine
- Ascorbic acid (vitamin C)
- Benzhexol (Artane)
- Chloramphenicol [high risk for Mediterranean/Asian variants]
- Chlorguanidine (Proguanil, Paludrine)
- Chloroquine
- Colchicine
- Diphenhydramine (Benadryl)
- Isoniazid
- L-Dopa
- Menadione sodium bisulfite (Hykinone)
- Menapthone
- *p*-Aminobenzoic acid
- Phenylbutazone
- Phenytoin
- Probenecid (Benemid)
- Procain amide hydrochlonde (Pronestyl)
- Pyrimethamine (Daraprim)
- Quinidine
- Quinine
- Streptomycin
- Sulfacytine
- Sulfadiazine
- Sulfaguanidine
- Sulfamerazine
- Sulfamethoxypyridazine (Kynex)
- Sulfisoxazole (Gantrisin)
- Trimethoprim
- Tripelennamine (pyribenzamine)
- Vitamin K
