# Supplementary material for: Glucose-6-phosphate dehydrogenase activity measured by spectrophotometry and associated genetic variants from the Oromiya zone, Ethiopia
Source: Malar J. 2018 Oct 12;17:358. doi: 10.1186/s12936-018-2510-3 (PMC6186078; doi:10.1186/s12936-018-2510-3)
Supplement: Supplementary file 4 — Additional file 4. G6PD Card (Amhara). [file 12936_2018_2510_MOESM4_ESM.pdf]

ግሉኮስ-6-ፖስፌት ድሃድሮጅኔስ (ግ6ፖዲ) ኢንዛይም እጥረት ካርድ

ስም:-____

የልደት ቀን :-____

አድራሻ:-____

ከላይ በስም ተጠቃሹ ግ6ፖድ ኢንዛይም እጥረት ____________________% አለው፡፡

ይህም ማለት መጠነኛ/የከፋ ኢንዛይም እጥረት፣ ምናልባት የአፍሪካዊ ልዩነት ሊሆን ይችላል፡፡ ምርመራው ጅማ ዩኒቨርስቲ ሆስፒታል በ ተሰር•ል፡፡

ግ6ፖድ ኢንዛይም እጥረት በዘር የሚተላለፍ የቀይ ደም ህዋስ በሽታ ነው፡፡ ተላላፊ በሽታ አይደለም ነገር ግን ህመምተኞች የተወሰኑ መድሃኒቶችን ሲወስዱ በጣም አደገኛ ነው፡፡ እነዚያ የተወሰኑ መድሃኒቶች ህልፈተ-ህይወት የሚያደርስ በሽታ (የቀይ ደም ህዋስ መጥፋትን) ያስከትላሉ፡፡

እንደዘር ልዩነታችን በሽታ (አፍሪካ፣ ኤስያ፣ሜደትራኒያን ል ዩነቶች)፣ የተወሰኑት መድሃኒቶች ከፍ ያለ ወይም አነስተኛ ጎጂ ናቸው፡፡

ስለዘር በሽታው መረጃ ኢንዛይሙን ከተመረመሩ 3 ወር በሁላ በ ዶ/ር ስንታየሁ ፍቃዱ ጅማ ዩኒቨርስቲ ላይ ሊሰጥ ይችላል፡፡

የሚከተሉት መድሃኒቶች መሰጠት የለባቸውም (አደገኛ)

አሴታሊኒሊድ (አሲታሊኒድ)	$C_8 H_9 N O$
አሴታይልፌናይልሃይድራዚን	$C_8 H_{10} N_2 O$
አልደሳለፎን ሶዲየም (ሳልፎክሶን)	$C_{14} H_{14} N_2 Na_2 O_6 S_3$
አረሲን	As-H3
ቤታ-ናፍቶል(2-ናፍቶል)	C10 H8 O
ክሎሮአምፊኒኮል	C11 H12 C12 N2 O5
ክሎሮኩየን	C18 H26 Cl N3
ሲፕሮፍሎክሳሲሊን	C17 H18 F N3 O3
ዳፕሶን (ዲአፌኒልሳልፎን)	C12 H12 N2 O2 S
ዲመረካፕሮል	C3 H8 O S2
ዶክሶሩቢሲን	C27 H29 N O11
ፉራዞሊዶን	C8 H7 N3 O5
ግሊቤነክላሚድ	C32 H28 Cl N3 O5 S
ግሉኮሳለፎን (ግሉኮሳልፎን ሶዲየም)	C24 H34 N2 Na2 O18 S3
አይሶቡታይል ናይትራይት	C4 H9 N O2
መናዲአል ሶዲየም ሳልፌት (ቫይታሚን ኬ 4 ሶዲየም ሳለፈዬት)	C11 H8 Na2 O8 S2
ሜናዳይኦን (ሜናፍቶን)	C11 H8 O2
ሜናዳዩኦን ሶዲየም ቢስልፊት (ቫይታሚን ኬ3 ሶዲየም ቢሳልፋይት)	C11 H8 O2 NaHSO3
ሜፓክሪን (ኩይናክራይን)	C23 H3O Cl N3 O
ሜሳላዚን-5-አሚኖሳሊሳይክሊክ አሲድ (ፓራአሚኖሳሊሳይክሊክ አሲድ)	C7 H7 N O3
ሜታሚዞል	C13H16N3NaO4S
ሜታይልቲይኦኒየም ክሎራይድ (ሜታይልን ብሉ)	C16 H18 Cl N3 S

ናልዲሲክ አሲድ	C12 H12 N2 O3
ናፕታሊን፣ፑዩር (ናፕታሊን)	C10 H8
ኒሪዳዞል	C6 H6 N4 O3 S
ኒትሮፉራል(ኒትሮፉራዞን)	C6 H6 N4 O4
ናይትሮፉራንሸን	C8 H6 N4 O5
ኦ-አስትልሳልሳሊክ ኣሲድ (አሲቲልሳልሳሊክ ኣሲድ)	C9 H8 O4
ኦክሲዴዝ፣ ዩሬት(ዩሬት ኦክዚዴዝ)	
ፓማኩይን	C42 H45 N3 O7
ፔንታኩይን	C18 H27 N3 O
ፌናሲቲን(አስቶፌንዲን)	C10 H13 N O2
ፌናዞፔሪዲን	C11 H11 N5
ፊኒልድራዚን	C6 H8 N2
ፕሪማኪዩን	C15 H21 N3 O
ፕሮቤንሲድ	C13 H19 NO4 S
ሰቲቦፊን(2-ኦክሲዶ-3-5-ዳሳልፎናቶፌኖክሲይ)-1፣3፣2፣ ቤንዞዲኦክሳሰተቦል-4-6-ዲሳልፎኔት	C12 H4 Na5 O16 S4 Sb
ሳልፋስታማይድ	C8 H10 N2 O3 S
ሳልፋድማይድ	C12 H14 N4 O2 S
ሳልፋፉራዞል(ሳልፋራዞን፣ሳልፊሶክዛዞል)	C11 H13 N3 O3 S
ሳልፋሜቶክዛዞል	C10 H11 N3 O3 S
ሳልፋኒላማይድ(ሳልፋኒልአማይድ	C6 H8 N2 O2 S
ሳለፋፕይሪዲን	C11 H11 N3 O2 S
ሳልፋሳላዚን፣ ሳላዞሱፋፕይሪዲን (ሳላዞፒሪን)	C18 H14 N4 O5 S
ዚኣዞሱልፎን (ዚአዞልሱለፎን)	C9 H9 N3 O2 S2
ቶሎኒም ክሎራይድ፣ ቶሎኒየም ክሎራይድ (ቶሉዲኒ ብሉ)	C15 H16 Cl N3 S
ተራኒተሮቶሉን(2፣4፣6-ተሪኒተሮሉን)	C7 H5 N3 O6

የሚከተሉት መድሃኒቶች አደጋቸው ዝቅ ያለ ነው፡፡

አሴታሚኖፍን (ፓራሲታሞል፣ታይኖል፣ትራልጎል
አሴቶፍንቲዲን (ፌናስቲን)
አሚኖፕራይን(ፓይራሚዶን፣አሚዶፓሪን)
አነታዞሊን
አንቲፓይሪን
አስክሮቢክ አሲድ(ቫይታሚን ሲ)
ቤነዝህክዞል
ክሎሮአምፊኒኮል (ለሜዴትራኒያን/ለአሲያ አደገኛ
ክሎሩጉአኒዲን (ፕሮጉአኒሊ፣ፓሉደሪን)
ክሎሮኩይን
ኮልቺሲን
ዳይፌንሃይደራማይን (ቤናደረይል
አይሶኒአዛይድ
ኤል-ዶፓ
ሜንዳይኦን ሶዲየም ቢስለፋይት (ሔኪኖን)
ሜናፍቶን
ፓ-አሚኖቤንዞይክ አሲድ

ፌኒልቡታዞን
ፌኒልቶይን
ፕሮቤንሲድ (ብንሚድ)
ፕሮኬይን አማይድ ሃይድሮክሎንድ
ፓይሪሜታሚን (ዳራፕሪም)
ኩይኒዲን
ኩይኒን
ስትሬፕቶማይሲን
ሳልፋሲይቲን
ሳልፋዲአዚን
ሳለፋጉኣንዲን
ሳልፋሜራዚን
ሳለፋሜቶክሲፓይሪዳዛን (ካይኔክስ)
ሳለፊሶክዛዞል (ጋንትሪሲን)
ትራይሜቶፕሪም
ትሪፔሌናማይን (ፕይሪብንዛማይን)
ቫይታሚን ኬ
