# Supplementary material for: Glucose-6-phosphate dehydrogenase activity measured by spectrophotometry and associated genetic variants from the Oromiya zone, Ethiopia
Source: Malar J. 2018 Oct 12;17:358. doi: 10.1186/s12936-018-2510-3 (PMC6186078; doi:10.1186/s12936-018-2510-3)
Supplement: Supplementary file 5 — Additional file 5: Table S1. G6PD activity in per cent and associated gender distribution. [file 12936_2018_2510_MOESM5_ESM.docx]

**Additional file 5: Table S1. G6PD activity in per cent and associated gender distribution**

| G6PD activity in % | All  n (%) | Male  n (%) | Female  n (%) |
| --- | --- | --- | --- |
| <70 | 0 | 0 | 0 |
| 70-80 | 11 (5.4) | 8 (4.9) | 3 (7.3) |
| 80-90 | 26 (12.8) | 24 (14.7) | 2 (4.9) |
| 90-100 | 68 (33.3) | 56 (34.4) | 12 (29.3) |
| 100-110 | 52 (25.5) | 41 (25.2) | 11 (26.8) |
| 110-120 | 33 (16.2) | 27 (16.6) | 6 (14.6) |
| 120-130 | 9 (4.4) | 4 (2.4) | 5 (12.2) |
| >130 | 5 (2.4) | 3 (1.8) | 2 (4.9) |
